# Exploring the Relevance of Indigenous Knowledges to Dementia Care in Nursing

**DOI:** 10.1111/nup.70018

**Published:** 2025-01-27

**Authors:** Christine Meng, Helen Brown

**Affiliations:** ^1^ School of Nursing, Faculty of Applied Science University of British Columbia Vancouver British Columbia Canada

**Keywords:** dementia care, embodiment, holism, indigenous knowledges (IKs), relationality

## Abstract

In this paper, we engage in philosophical inquiry to consider the relevance of Indigenous Knowledges (IKs) for reimagining dementia care for individuals living with dementia. We outline the limitations of philosophical perspectives aligned with Eurocentric academic knowledge, arguing that such knowledge relies on an individualistic view of self and neglects the body and embodied experience in dementia care. We demonstrate how a personal diachronicity perspective diminishes the importance of valuing the fluid and dynamic self‐identities of persons living with dementia. We then turn to the epistemological foundations of IKs through philosophical inquiry, focusing on relationality, connectiveness, and holism, and discuss the role of IKs in institutional knowledge systems. We then explore the potential relevance of IKs to widen the epistemological and ontological gaze centering on the relational concepts of personhood, holism, continuity, embodiment, and homogeneity of self that are foundational to reimaging dominant approaches to dementia care.

Over the years, scholars have investigated various philosophical and theoretical perspectives aimed at improving the quality of life of individuals living with dementia. These include person‐centred care (Kitwood 1998) and, more recently, relationship‐centred care (Engel, Pethtel, and Zarconi 2018). Person‐centred care acknowledges every individual's right to dignity, respect, and full engagement with their surroundings, emphasising personal values, histories and personalities (Kitwood 1998). Relationship‐centred care highlights the importance of relationships between individuals, family caregiver, health care professionals and the community in health and healthcare deliver, promoting mutual recognition of reciprocal connections (Engel, Pethtel, and Zarconi 2018).

Despite the diversity of approaches in dementia care, these care approaches are generally informed by knowledge produced in Eurocentric academic health sciences (Kim and Park 2017; Morhardt and Spira 2013; Nwadiugwu 2021). A growing area within nursing and health sciences, particularly in the context of a global crisis, is indigenous caregiving (Racine et al. 2022). To support informal caregivers and adjust care strategies, researchers must pay close attention to Indigenous values and voices (Racine et al. 2022). This has influenced efforts to broaden the dominance of Eurocentric academic health sciences in dementia care by making space for qualitatively derived knowledge in the academy, introducing subjectivity and multiple truths into epistemological spaces. In this paper, we use the term ‘Eurocentric academic health sciences’ in this paper to reflect an epistemological orientation to knowledge historically rooted in positivism and post‐positivism. However, we recognise that there is considerable diversity within the epistemological foundations from which knowledge is derived within Eurocentric academic health sciences.

Eurocentric academic health sciences have, for the most part, been derived from positivist thought traditions, where the pursuit of general laws or principles through ‘objective’ observation is the aim of knowledge generation (Smylie, Olding, and Ziegler 2014). In the positivist tradition, knowledge is understood to exist independently of the individuals or locations from which it originates and may be measured and tallied (Guba and Lincoln 1994). The dominance of Eurocentric knowledge in academic health education and practice, which can be traced back to colonialism in the Canadian context^1^ has both erased and marginalised Indigenous Knowledges (IKs), which have existed for thousands of years (Toivonen, Charalambous, and Suhonen 2018). In nursing, such marginalisation limits the diversity of perspectives crucial for addressing complex healthcare challenges (Stansfield and Browne 2013). Informed by the scholarship of Indigenous scholar Cash Ahenakew (2021), we employ IKs to frame knowledge as a pluralistic concept. This approach acknowledges the unique and diverse knowledge systems among Indigenous communities, emphasising that there is no singular ‘Indigenous knowledge.’ Consequently, the term is not intended to suggest interchangeability among the distinct knowledge systems of various groups, nor to diminish the profound diversity inherent to Indigenous communities.

In this paper, we consider the role that IKs can play in shaping and influencing the range of knowledge necessary for providing nursing care for individuals living with dementia, and how perspectives grounded in IKs can expand knowledge for nursing practice within the context of dementia care. We begin by situating ourselves as authors within this discourse, describing our social location and perspective. We then critically examine how philosophical perspectives aligned with Eurocentric academic health sciences can be expanded to better incorporate Indigenous epistemologies. We argue that the prevailing focus on an individualistic self in Eurocentric academic health sciences often renders the embodied experience of dementia care less visible^2^, overlooking the fluid and dynamic identities of those living with dementia. Next, we explore the epistemological foundations of IKs using philosophical inquiry, focusing on relational views, connectedness, and holistic understanding. We also briefly discuss the role of IKs in institutional knowledge systems within academic contexts to connect our inquiry here to the expanding efforts for decolonisation within Canadian universities, using tenure and promotion as an example. Our discussion then turns to the potential relevance of IKs to widen the epistemological and ontological gaze to centre the relational concepts of personhood, holism, continuity, embodiment, and homogeneity of self that are foundational to reimagining dominant approaches to dementia care.

## 1 Positionality and Background

The first author acknowledges their positionality as a woman of colour and first‐generation immigrant living, working, and playing in the unceded homelands of the Musqueam, Squamish, and Tsleil‐Waututh people in Vancouver, BC. The second author is a settler of mixed European ancestry also working and living on the unceded, occupied, traditional and ancestral territory of the Coast Salish Peoples, including the territories of xʷməθkwəy̓əm, Sḵwx̱wú7mesh, and Səl̓ílwətaɬ. The second author, has been co‐leading cultural safety, equity, and decolonisation work within the School of Nursing and has been deeply engaged in participatory research with Indigenous communities for over 15 years where IKs guide all processes, protocols and methodologies. In our respective work, we both aim to extend beyond the individualistic concepts of identity generated from positivism and post‐positivism, critically reflecting on how positionality and power in a broader socio‐cultural context shape social location and identity. As the first author, I will provide additional in‐depth reflection as a doctoral student to inform my ongoing development as a critical researcher committed to decolonising academic spaces. This approach aims to pave the way for greater epistemological pluralism in nursing and dementia care, thereby aligning with the IKs concept of understanding the world through multiple lenses.

As mentioned earlier, the first author's identity is constituted through multiple positionalities as both a woman of colour and a first‐generation immigrant. This identity has enabled an understanding of the power dynamics that sustain experiences of marginalisation in a complex social world. Power refers to the ability or potential to influence and affect the behaviour of others, and power dynamics revolve around positions of power (Crosschild et al. 2021). Different sources of power exist within power dynamics. Positional power is the authority that an individual in an organisation's hierarchy and structure is believed to have due to their position, which is frequently influenced by ‘norms’ and values (Northouse 2021). Applying this power dynamic to the first author's identity, it is recognised that objective realities, such as violence and discrimination, are forms of power. Additionally, power can stem from the inherent position of power, where dominant cultures can shape and regulate knowledge through control over economic and material allocation of resources.

Considering multiple identity positions can be further understood by turning to Patricia Hill Collins's work on intersectionality that prompts examination of how a person's experiences and social status are shaped by the intersection and interaction of numerous social identities and oppressive institutions (Collins and Bilge 2020). The concept of intersectionality recognises that individuals can possess multiple social identities, including race, gender, class, sexual orientation, and disability, and that these identities cannot be understood independently of one another. Instead, they overlap and mutually influence one another, giving rise to complex and individual experiences of privilege and oppression (Collins and Bilge 2020). This perspective is helpful here wherein we consider the intersection between our positionalities as nursing academics who have been shaped by Eurocentric epistemologies and our commitments to expand forms of knowledge that can contribute to improved nursing care for people living with dementia. We now turn to a discussion of how Eurocentric concepts of identity and personhood in dementia care impact nursing care, including the examination of quality of life for individuals living with dementia.

## 2 Eurocentric Concepts of Identity and Personhood in Dementia Care

Eurocentric philosophical perspectives derive from an individualistic concept of the self, particularly within the context of individuals living with dementia. For example, from the dualist Cartesian perspective, an individual with dementia is viewed as being mindless because their mind is considered absent or nonexistent (Dewing 2008). Dewing (2008) argues that this leads to neglect of the body and embodied experience, resulting in what he terms the *empty‐shell view of dementia*. Based on divisive understandings of the brain, mind, and body, the body from this perspective is only acting as a carrier. Given the value in Eurocentric academic health sciences ascribed to normative brain function, particularly memory, what is left when dementia ‘shatters’ brain and mind functioning—the empty shell—is regarded as having little worth. While people living with dementia change from the selves they were before the disease onset, the natural consequence ought not consider them as inferior simply because those around them perceive to be less than functional human (Dewing 2008). He describes a shift in the interpersonal relationships and social positions of people with dementia from an ‘I–thou’ to ‘I–it’ dynamic, where the ‘it’ reflects an object, no longer regarded as a subject.

With respect to the identity of self of an individual living with dementia, health care professionals regard a match between mind and the body in a single linear order as being a desired outcome of our interventions. If a 95‐year‐old woman living with dementia, were to ask a nurse to call her mom to come pick her up, health care professionals conclude that the brain of an individual living with dementia has ‘shattered’ and that it is now an empty shell disconnected from the body. Health care professionals also perform medical interventions based on this view of reality. Health care professionals ask family members to bring in old photos to help restore the individual living with dementia to our own reality. But such actions can often cause more harm than good; for example, if the individual living with dementia is simply unable to become oriented to reality in the way family or health care professionals hope and only becomes frustrated and confused.

The humanity and personality traits that persist in a person with dementia are neither valued nor recognised. Gerontological literature frequently highlights that the behaviour, activities, and conversations of persons with dementia are not always acknowledged, particularly when their behaviour is deemed problematic (Trahan et al. 2011; Trivedi et al. 2019).

## 3 Personal Diachronicity in Dementia Care

From a Cartesian perspective, it is worth considering whether it is possible to comprehend the nature of one's self and identity without turning to external knowledge based on one's physical body (Higgs and Gilleard 2016). Treating ‘self’ and ‘personhood’ as separable from the material body further reinscribes dominant disembodied concepts of self and identity that underlie dementia care. From a Eurocentric academic health science perspective, the self and personhood are seen as independent of the body, and hence not reducible to the fundamental or primary particulars such as matter (Higgs and Gilleard 2016). If the self, the body, and personal identity are considered components of a person, then acknowledging one's states of consciousness as a unique and generative aspect of self can expand the gaze for relevant knowledge that promotes dignity and holistic wellbeing for individual living with dementia. While states of consciousness have unity, they naturally may fluctuate in time and form (Higgs and Gilleard 2016).

To further illustrate how Eurocentrically derived personal diachronicity operates in dementia care, consider the assessment instruments used to measure quality of life (QoL) for individual living with dementia. Due to the decline in cognitive function brought on by dementia, QoL is often reported by proxies such as family members or medical professionals (Rabins et al. 1999; Thorgrimsen et al. 2003; Weiner et al. 2000). Yet, individuals with moderate to severe dementia have the ability to rate their own QoL, despite the fact that proxy rated QoL (PRQoL) is the frequent measure used in dementia care. Although many studies rely solely on proxies to report QoL, Burks et al. (2021) have found that doing so might not provide a complete picture of what comprises QoL. It has been observed that family members' assessments of an individual's QoL do not align with those of the individuals themselves, and health care providers frequently underestimate the individual's QoL (Katsuno 2005). The PRQoL has consistently ranked lower than SRQoL in studies that suggests individual living with dementia believe they have greater QoL than is generally reported in the literature, which may provide comfort to those who are caring for people living with dementia (Moyle et al. 2012). While proxy reports should still be considered, it is important to recognise that there may be a bias toward reporting negative behaviours, such as irritation and depression, as having a greater impact on QoL than perceived by the individuals with dementia (Burks et al. 2021).

Self‐reported QoL ratings tend to use the individual's subjective feelings and mood states as their primary reference point, unlike proxy QoL ratings (and the measures intended to gather them), which heavily emphasize the observable and objective aspects of the person with dementia's current functioning and performance. This latter finding ties self‐reported evaluations by people living with dementia to deductive (‘top‐down’) theories of subjective well‐being found in the general population. These theories contend that self‐reported QoL ratings reflect a person's overall emotions of well‐being rather than being primarily based on a systematic evaluation of particular performances, events, and experiences. In other words, QoL assessments are more influenced by individual attitudes and personality factors, rather than by the alternations of changing circumstances (Ormel 1983; Trigg et al. 2012). The limitations observed in the development of proxy‐rated quality QoL assessments serve to further reinforce the influence of personal diachronicity rooted in Eurocentric academic health sciences. These assessment criteria entail a logical and empirical necessity to overlook the significance of valuing the flexible self‐identity within the inevitable transformative process of the physical and mental constituents of individual living with dementia.

## 4 Orienting to Indigenous Knowledges

Indigenous researchers emphasize how IKs entails much more than the binary opposites of Eurocentric academic health sciences. IKs consists of the philosophies, heritages, and educational systems of Indigenous peoples, deeply considering relationality and interconnectedness that fills ethical and epistemological gaps in Eurocentric scholarship including its theories, methodologies, evidence, and conclusions (Battiste 2002). The term ‘Coming to know’, which implies a journey, refers to the process of creating or discovering Indigenous ways of living in nature (Cajete 1994). This is distinct from the Eurocentric scientific process of knowing, which denotes a final result, such as a patent or a published record of a discovery (Aikenhead and Ogawa 2007). In Eurocentric academic health sciences, knowledge translation is typically viewed as the transfer of expert knowledge from researchers to health care practitioners, characterised by knowledge specialization and academic silos (Smylie, Olding, and Ziegler 2014). By contrast, Indigenous ‘coming to know’ is not a destination of discovery but a journey toward wisdom or a path of wisdom‐in‐action (Aikenhead and Ogawa 2007).

In Eurocentric academic health sciences, the prevailing, hegemonic view of ‘the scientific method’ tends to make assertations about reality by assuming that there is only one reality discoverable through scientific procedure. In this view, the purpose of science is to draw inferences about reality by providing evidence about it through scientific research; only knowledge that satisfies the criteria of objectivity established by scientific investigation is regarded as valid, while other representations or modes of understanding the world are regarded as less valid (Martin 2012). We argue, as many other have, this paradigm limits the potential values and relevance of other knowledge sources or the relationships between different forms of knowledge for nursing in specific contexts.

Moreover, Harding (2015) argued that Eurocentric academic health sciences have appropriated concepts and customs from other cultures, which are often exploitative. The intentional destruction of the societies and environments that support IKs systems has frequently occurred as a result of colonial or imperial violence (Harding 2015). Consequently, maps of various types of purportedly natural social relations are always present in these sciences as well, as social relations themselves are influenced by beliefs and behaviours that advance and validate modern Eurocentric academic health science sciences while devaluing IKs systems (Harding 2015). The societies of the modern West are thoroughly incorporated inside their respective disciplines and vice versa (Harding 2015).

According to Cajete (1994), IKs covers a wide spectrum of learning processes that emerge from interactions between human beings and the natural world. IKs is founded on the assumption that all bodies and minds, being manifestations of a more profound spiritual essence, are interconnected (Sheridan and Longboat 2006). As a component of the wider web, human consciousness can be aware of the cosmic link. Given that everything in the universe is interconnected, information obtained through these connections entails a responsibility on the part of the knowledge‐keeper (Hatcher et al. 2009). From an Indigenous point of view, it may seem self‐centred for a person to possess knowledge but not use it in their daily lives or share it for the benefit of the community (Smylie, Olding, and Ziegler 2014). Two Canadian Indigenous scholars, Battiste (2002) summarize the structure of IKs as follows:

(a) knowledge of unseen powers in the ecosystem; (b) knowledge of the interconnectedness of all things; (c) knowledge of the perception of reality based on linguistic structure or ways of communicating; (d) knowledge that personal relationships bond people, communities, and ecosystems; (e) knowledge that traditions teach specialized knowledge related to morals and ethics; and (f) knowledge that extended kinship passes on social traditions and practices from one generation to the next

(p. 143).

We see it as critical to emphasize that fully engaging with the complex and diverse conversations and scholarly dialogue related to IKs in both academic and practice contexts is unattainable within a single article. Yet, we are committed to ensuring this conversation continue as a result of our focus on IKs and dementia care. Cree researcher Shawn Wilson (2001) explains that an Indigenous worldview ‘originates from a fundamental belief that knowledge is relational’. This perspective emphasizes how knowledge encompasses both relational and individual aspects (p. 176). Unlike Eurocentric knowledge, learning about Indigenous ways of life in the natural world involves experiencing procedures similar to those of Indigenous lifestyles (Aikenhead and Ogawa 2007). Understanding how to live in the natural world requires more than merely reading an article. Indigenous ways of life are action‐oriented and verb‐based; they cannot be bestowed, summed up, banked, or evaluated using paper‐and‐pen tests (Aikenhead and Ogawa 2007). They must be experienced within the context of numerous relationships, by living in a specific natural setting, and through seeking enlightenment (Aikenhead and Ogawa 2007). In the following section, we discuss the role of IKs in institutional knowledge systems, particularly focusing on their use in academic contexts related to colonial endeavours such as tenure and promotion.

## 5 The Role of Indigenous Knowledges in Institutional Knowledge Systems

It is widely recognised that community‐based work is time‐intensive. Faculty members engaged in community‐based participatory research often struggle to balance the additional time this research approach demands with their other academic obligations (Riley et al. 2023). Consequently, a tenure‐track woman of colour who commits to conducting ethnographic research and interpretive writing, rather than opting for quicker, more widely accepted conceptual publications, may encounter significant challenges in securing tenure and promotion, endeavours often influenced by colonial biases (Pierce 2013). Indigenous faculty members can find themselves making decisions that conflict with their axiological commitments, questioning their methodological choices, and restricting their freedom of expression, given the high stakes of tenure (Council of Ontario Universities 2020). Ellingson and Quinlan (2012) urge universities leaders to consider a variety of ‘products’ in their hiring, evaluation, tenure, and promotion processes. Similarly, Louie (2019) questions whether IKs could play a role, even partially, in the hiring process. For instance, presenting at conferences provides academics with opportunities to expand and disseminate their knowledge. Recognizing rituals, communal activities, and obligations essential for scholarly development alongside institutionally approved conferences and distribution sites would represent a progressive step (Louie 2019). Implementing additional policy changes concerning research, education, and service could potentially broaden the tenure and promotion frameworks within universities (Louie 2019).

According to Louie (2019) the importance of maintaining relationships may appear trivial to the typical emphasis on publishing and securing research grants as metrics of academic accomplishments. Thought‐provoking questions such as ‘Who am I?’, ‘Where do I come from?’, ‘Where am I going?’, and ‘What obligations do I hold?’ are sometimes dismissed as irrelevant to meritocracy and academic accomplishments towards tenure and promotion. Before researchers can establish partnerships and engage with IKs and community partners effectively, they must understand how academic colonialism has and continues to shape mistrust and harmful processes to emphasize the critical important of building local relationships, becoming trustworthy, learning and following local protocols and being skilled at knowing how to value multiple sources of knowledge and expertise (Varcoe et al. 2011). Even after research concludes, the researchers' obligations continue. Otherwise, communities may perceive the partnership as merely a facade to secure research subjects (Louie 2019).

Building relationships is crucial to the success of academic‐Indigenous community partnerships (Riley et al. 2023). However, overly complex institutional procedures, while intended ensure ethical relationships, have complicated faculty members' ability to interact as expected with these communities (Riley et al. 2023). Though vital for maintaining connections long after projects end, such efforts are frequently overlooked in annual performance reports and tenure applications, although they are emphasized in recruitment advertisements that mention ‘Indigenous Knowledges’ (Louie 2019). Ultimately, the restrictive definition of ‘peer‐reviewed publication’ as indicators of academic impact can impact career progress. For example, Riley et al. (2023) indicated that Indigenous faculty members are often forced to choose between meeting publishing requirements and fulfilling their community service duties, potentially jeopardising their tenure and thereby their future capacity to move through the stages of building their academic career for promotion.

In the section that follows, we illustrate one approach to integrating IKs and Eurocentric academic health sciences, Two‐Eyed Seeing, to consider the relevance for nursing and caring for individual living with dementia.

## 6 Two‐Eyed Seeing

In 2004, Mi'kmaq Elders Albert and Murdena Marshall of Unama'ki (Cape Breton), Nova Scotia, Canada, proposed the concept of Two‐Eyed Seeing, which emphasizes the value of seeing the world from both Western and Indigenous perspectives (Wright et al. 2019). Originally developed to help Mi'kmaq university students engage in their science studies, the concept of Two‐Eyed Seeing extends beyond Mi'kmaq worldviews, blending the advantages of both mainstream (Western) and Mi'kmaq knowledge. Because of the binocularity of this guiding concept, integrative research benefits from a larger, deeper, and more creative field of vision than can be achieved by either viewpoint alone. Two‐Eyed Seeing does not merely combine two distinct knowledge systems or overlay portions of Indigenous and Western knowledge (Reid et al. 2021). Nor does it imply that one should view the world in only two ways. The idea of the metaphor is to emphasize a holistic approach: using both eyes to observe the world in an integrated way. Those with differing worldviews must value working together to create knowledge and value learning from and appreciating the differences of others. There is no hierarchy of knowledge or of ways of knowing in Two‐Eyed Seeing. Instead, in this view individuals should take a holistic approach that embraces mind, body, and spirit, and should reflectively examine their own perspectives, beliefs, and values. The analogy of trees holding hands (intertwining their roots) is used to describe this way of joining with one another despite people's differences (Wright et al. 2019).

Two‐Eyed Seeing empowers researchers to adopt a decolonised research plan through critical reflexivity. A decolonised research plan necessitates careful reflection of the influence of colonisation on the current expression of IKs as well as the ways in which colonial experiences have shaped IKs (Martin 2012). Therefore, decolonised Indigenous scholarship does not presuppose that a state of pre‐colonisation can ever be achieved (Martin 2012). However, by considering how colonisation has shaped IKs, researchers can start to recognise colonial practices and move past the boundaries it has established, rejecting forms of knowledge that further a colonial agenda (Martin 2012). This understanding leads to discussion of positionality and power between IKs and Eurocentric academic health sciences. Although positionality and power are inherent in all research studies, Crosschild et al. (2021) argue that a critically reflexive approach can more effectively address power dynamics, language use, and Eurocentrism in academic.

Critical reflexivity provides a platform for critical reflection not only on oneself but also on partner relationships, health institutions, and other interpersonal interactions (Rix, Barclay, and Wilson 2014). It enables monitoring the reciprocal workings of power, including the researcher's evolving position within the research process, facilitating a more comprehensive understanding of positioning, power, and inequity (Crosschild et al. 2021).

The concept of Two‐Eyed Seeing provides a framework for understanding how different types of knowing can coexist and be integrated through critical reflections. Though the lens of Two‐Eyed Seeing, rather than using a ‘microscope’ to comprehend populations of interest, research has a tendency to only use a ‘mirror’ to reflect on the role of the researcher in conducting research (Martin 2012). It suggests that health researchers in particular have a responsibility to look beyond what might be considered the ‘expertise’ of the research and consider how Indigenous communities have been alienated from the traditional ways of living and healing that have contributed to and ultimately 'caused' the health issues being experienced (Martin 2012).

In the section that follows, we explore epistemological foundations of IKs through philosophical inquiry, focusing on relational views, connectiveness, and holistic understanding. We also highlight the diversity and complexity of knowledge‐sharing practices among Indigenous communities.

## 7 Understanding of Connectiveness in Indigenous Knowledges

As noted by Reid et al. (2021, p. 245), for Murdena Marshall connectiveness is ‘the understanding of a person's relationship to the Creator and all of creation’. According to Reid et al. (2021) the three letters *‐ive* in ‘connectiveness’ convey a sense of activity, of leaning toward a state, especially in a regular or lasting way; the prefix *‐ness* adds the sense of state or intensity of the action. Since Mi'kmaq is a verb‐based language, the word ‘connectiveness’ distinguishes between the condition of being connected and the action of connecting (Reid et al. 2021). In Two‐Eyed Seeing, connectiveness is not simply the final product of a relationship; it is about the value of being in the moment to connect, about actively establishing ways of moving toward being connected through self‐reflexivity. According to Reid et al. (2021, p. 201), connectiveness is ‘inherent in all holistic subjects or holistic practices’ and closely aligns with interdisciplinary or transdisciplinary approaches in in Western scholarly discourse, explaining its compatibility with theories from cognitive science, complexity theory, and complexity science. Connectiveness represents a boundaryless way of accessing knowledge. There are no distinct criteria for assessing how knowledge or ways of knowing fit into the realm of Two‐Eyed Seeing or for identifying the conditions under which Two‐Eyed Seeing can grow. Two‐Eyed Seeing nurtures continuity and homogeneity in adapting knowledge or ways of knowing in order to understand the world.

Discussing holistic understanding within IKs, Battiste and Youngblood (2000) highlight the importance of a holistic approach to achieving harmony, defined as a multidimensional and dynamic balance of interrelationships within the ecologies of Indigenous peoples. Discord arises when these interrelationships are disturbed (Battiste and Youngblood 2000). Health care professionals challenge the principle of holism when they divide and categorise natural phenomena into biology, chemistry, and physics (Aikenhead and Ogawa 2007). Giroux (1992) also pointed out that beyond challenging the structure and content of Eurocentric sciences, a monist ontology and a holistic epistemology reproduce ‘new forms of knowledge through [their] emphasis on breaking down disciplines and taking up objects of study that were unrepresentable in the dominant discourses of the western canon’ (Giroux 1992, p. 56).

Holism can also mean integrating epistemological diversity and fostering interdisciplinary consensus in fields such as dementia care. According to Weigand (2011), individual disciplines—including linguistics, biology, sociology, and neurology—can no longer isolate themselves from one another. Instead, they form an integrated whole, where the actions and behaviours of people are not made up of discrete components. Interdisciplinarity involves awareness of activities across disciplines from the outset, rather than merely incorporating elements from other fields into an already been developed framework within a single discipline. Weigand (2011) argues that if people embrace consilience or the unity of knowledge, this understanding should guide current practices, not just future plans. The following metaphor illuminates a holistic perspective from within an IKs perspective:

A fish is not a river, and a river is not a fish. However, to understand a river, one must understand the ecology of fish. And, no one can study fish without knowing a great deal about the river that they live in. To explain these deep ecological relationships, our ancestors used stories; oral tradition. Of course, experiencing the physicality of the fish and the river is also required for knowledge of the two entities. Beginning to think this holistic way about reality/science can soften and dissolve the concrete walls between categories that enforce dangerous binaries in our present world. Such a move toward this holistic mind also implicitly re‐purposes both science and education more broadly.

(Marker 2016, p. 479)

In summary, IKs provide essential frameworks for incorporating cultural, spiritual, political, and social norms into the development and implementation of healthcare practices and evaluations. Assessing only one aspect of a person‐centred setting such as the physical indicators for QoL is limited and can be compared to knowing only the skeleton of a person rather than the whole person. The systems comprising people, places, policies, and social norms that shape these settings are dynamic, multi‐component, and interact on many different levels (Calkins, Kaup, and Abushousheh 2022). The following paragraphs further discuss the use of IKs and philosophical inquiry to reimagine dementia care for individuals living with dementia, focusing on continuity, homogeneity, and relationality.

## 8 Continuity and Homogeneity of Self in Dementia Care

Concepts of personal identity and the self‐have long been essential themes in metaphysics (Higgs and Gilleard 2016). Charles Taylor (1992) explored these concepts within the Western intellectual tradition, distinguishing between an inner ‘me’ and an exterior world composed of objects that are not ‘me’. A historically constrained manner of self‐interpretation is prevalent in the Eurocentric academic health sciences. As noted earlier, the Cartesian perspective distinguishes between knowledge obtained from cogito (internal thoughts) from knowledge obtained from the external material world and its quasi‐mechanical workings (Taylor 1992, p. 156). Consequently, healthcare professionals often find it problematic when individual living with dementia cannot align their identities within these dualistic frameworks. This challenge is rooted in Cartesian dualism, which views the mind and body as having separate functions (Baker and Morris 2005).

Reflection on the care of a 95‐year‐old woman living with dementia, whom we previously discussed in the article, brings to light several crucial aspects that warrant consideration. Instead of enforcing a conventional alignment of mind and body, healthcare professionals can use IKs to support individuals living with dementia as they develop more continuously and homogeneously within the context of their evolving identities. By recognizing that the previous identity may have been transcended, healthcare professionals can encourage family members to foster meaningful connections with the individual living with dementia, acknowledging their current state. Through the lens of IKs, healthcare professionals do not view the connection between mind and body as destroyed if people living with dementia cannot align their identity with conventional realities. Rather, they recognise that there is no single, appropriate combination of mind and body. Healthcare professionals embrace the notion that self‐conceptions and identities will evolve in a holistic way throughout the progression of the disease.

## 9 Centering Relationality in Dementia Care

Kitwood (1998) argues that dementia is best understood as the result of an interaction between neurological dysfunction and psychosocial factors, including health, personal psychology, and the environment, with particular emphasis on social context. He suggests that a person's abilities are as influenced by their environment as they are by their brain. Kitwood criticises the conventional medical approach to dementia, which primarily focuses on rigidly treating the illness, arguing that this traditional perspective often leads to negative consequences for caregiving (Fazio et al. 2018). In contrast, employing a holistic perspective from IKs in implementing person‐centred care for individuals living with dementia enables us to create a more comprehensive care model.

Consider, for example, a person living with dementia in a nursing home who informs a nurse that he is on duty tonight as a police officer despite having retired from police force ten years earlier. Healthcare professionals using conventional standards might classify this interaction as unusual and consider the treatment ineffective. They tend to perceive people living with dementia as existing within a single reality, inconsistent with other realities, and often establish standards and assessments based on what they consider norms without acknowledging the relational links to the history of the person living with dementia that underly his behaviour.

However, within the context of IKs (particularly a relational view of the challenge of the identity of self), emphasis is not placed solely on the outcome of an interaction. This relational view values the importance of being present in the moment to connect, actively seeking to establish connections with a person's past, present, and future. Instead of deeming a treatment ineffective because of an ‘incorrect’ outcome, healthcare professionals who embrace this approach acknowledge multiple realities. They assist person living with dementia in building connections with their past, present, and future selves. From this perspective, that the individual living with dementia is still on duty as a police officer can be seen as natural behaviours, and the intervention can be considerate as effective.

## 10 Conclusions

In conclusion, the authors highlight the relevance and potential of incorporating IKs into dementia care. IKs offers a holistic and inclusive approach that can significantly improve the quality of life for the population living with dementia. By integrating epistemological and ontological stances from IKs with those of Eurocentric academic health sciences, the authors argue that embracing multiple worldviews can enhance health outcomes for people living with dementia.

Additionally, the authors point out the limitation of the current knowledge base in dementia care, which is predominantly informed by Eurocentric academic health sciences. The individualistic orientation of such worldviews, where self and personhood are disembodied, restricts the diversity of perspectives and approaches that could otherwise foster the well‐being of individual living with dementia. By exploring the epistemological foundations of IKs and the concept of ‘Two‐Eyed Seeing’, the authors illustrate how incorporating Indigenous perspectives can enrich and broaden nursing approaches.

The primary goal of this paper has been to contribute to philosophical inquiry toward epistemological pluralism in nursing and academic institutions. This involves deep reflection on epistemological and ontological assumptions framed through Eurocentrism. Our aim has also been to actively create meaningful connections and engage in ongoing inquiry into diverse ways of knowing and to enhance holistic and relational centred care for people living with dementia.

## Conflicts of Interest

The authors declare no conflicts of interest.

## Data Availability

The authors have nothing to report.

## Endnotes

1 As noted by Indigenous scholars, the academic space continues to afford superiority to Eurocentric knowledge that has left little space for the valuing and centring of the potential contributions of IKs for nursing practice.

2 While nursing has a long tradition in the research, theory, and practice of integrating a phenomenological perspective that emphasizes the embodied concept of the self (Norlyk et al. 2023; Paterson and Zderad 1976), the uptake of this perspective in dementia care within nursing has lagged behind other areas of nursing practice. While studies are emerging, our aim is to incorporate the works of Indigenous scholars, considering embodied personhood, to build upon existing research and disrupt the colonial dominance of Eurocentric knowledge. This dominance has erased the value of IKs for dementia care in the nursing context.
